# PRIO – a prospective integrative oncology registry: trial protocol

**DOI:** 10.3389/fonc.2024.1455281

**Published:** 2024-12-10

**Authors:** Paul G. Werthmann, Ann-Kathrin Lederer, Hannah Maja Figura, Klaus Kramer

**Affiliations:** ^1^ Research Group Integrative Medicine, Department of General and Visceral Surgery, University Hospital Ulm, Ulm, Germany; ^2^ Center for Complementary Medicine, Department of Medicine II, Medical Center—University of Freiburg, Faculty of Medicine, University of Freiburg, Freiburg, Germany; ^3^ Institute for Applied Epistemology and Medical Methodology, University of Witten/Herdecke, Freiburg, Germany; ^4^ Department of General, Visceral and Transplant Surgery, University Medical Center of the Johannes Gutenberg University, Mainz, Germany

**Keywords:** integrative oncology, oncology registry, demography, quality of life, user profile, integrative oncology consultation

## Abstract

**Background:**

Integrative Oncology (IO) – the use of lifestyle modifications, mind-body practices and natural products in oncology – is increasingly asked for by patients. The evidence base of IO is growing and IO measures are included in national guidelines. Still, many questions about IO remain unanswered or still show a poor evidence base.

**Method:**

Data about demography, socioeconomic status, cancer disease and therapy, integrative oncology measures and patient reported outcomes will be collected in regular visits in oncological patients at the University Cancer Center Ulm, Germany. An expansion to further study centers is planned. After one year and at regular intervals, the registry will be evaluated and adapted accordingly.

**Discussion:**

The PRIO registry builds a solid data base to evaluate the use of integrative oncology measures in cancer patients. It includes patient reported outcome measures to investigate quality of life and satisfaction with health services in this population. The registry aims to enhance transparency in IO use and wants to inform future research in IO. The trial has been registered in the German Clinical Trials Register (DRKS) under the ID DRKS00033250 and in the studyBox of the German Cancer Association under the ID ST-U173 on 18th December 2023. The trial was approved by the ethics committee of the University Medical Center Ulm under the number 375/23 on 7th December 2023.

## 1 Introduction

Integrative Oncology aims to integrate lifestyle modifications, mind-body practices and natural products into the therapy of cancer patients undergoing guideline defined cancer treatment. With these measures it wants to contribute to the well-being of oncological patients, alleviate symptoms from the disease or tumor therapy, increase the body’s resilience and help in coping with the changes through the disease (1). Integrative Oncology measures (IOM) are seen as a “good supplement to standard cancer treatments” by 73% in the 2020 National Cancer Opinion Survey conducted by the American Society of Clinical Oncology [ASCO (2)]. IOM are increasingly asked for by patients. Newer developments within the field show an increasing evidence base of IOM and an increasing integration of IOM into cancer care. In 2018 the ASCO endorsed the breast cancer guideline of the Society of Integrative Oncology [SIO (3)]. Currently a joint guideline of SIO and ASCO followed for integrative oncology measures in oncological pain management (4). In Germany, a *S3-Guideline Complementary Medicine in the Treatment of Oncological Patients* was published in September 2021 (5).

Integrative oncology is a field in motion, with new approaches and interventions emerging, new trends and new developments. Although the evidence base of IOM is increasing, many questions remain unanswered or still show a poor evidence base (6, 7): How many patients use IOM? Which measures are used? Are there specific IOM that are employed with such frequency that further investigation would be advantageous? Do users of IOM have special demographic, socioeconomic or life style properties? Do they have a better quality of life and satisfaction and do they achieve better symptom control? Many patients do not report the use of IOM, which can also have negative consequences for the therapy (8, 9). This leads to questions such as: Do patients in conventional oncology trials use IOM, and do they introduce bias into oncology research if undetected? With these questions in mind we want to establish a registry of cancer patients’ use of IOM.

The *Prospective Integrative Oncology Registry* (PRIO) is a prospective explorative observational registry study. It is intended for cancer patients treated at the comprehensive cancer center Ulm (CCCU). The expansion to further partners in Germany and later on an international level is planned. The applied treatment is based on national guidelines according to tumor board decision/resolution with additional licensed integrative medical treatment. PRIO wants to enhance the evidence base of cancer patients using IOM regarding (i) the demographic, oncologic and life style properties of these patients, (ii) the integrative oncology measures they use, (iii) their quality of life and satisfaction and symptom control. The registry can detect correlations between certain interventions and health outcomes. Clinical efficacy studies could be nested within the current design as randomized clinical intervention trials in the future.

## 2 Methods

Patients will be recruited at the CCCU. Patients with all types of cancer will be included.

We enroll patients over the age of 18 who have been diagnosed with cancer and treated at the CCCU with written informed consent and the ability to understand the nature and individual consequences of the clinical trial. Exclusion criteria are a clinically significant concomitant disease critically influencing the ability of the patient to protocol adequate behavior or any finding that, in the judgment of the study investigator, renders the participant unsuitable for the study protocol.

Patients are free to withdraw from the study at any time without having to justify their decision. When a trial participant withdraws his/her informed consent, he/she is asked to decide whether the data captured so far may be kept in the data bank of if it has to be removed and deleted.

The trial may be prematurely closed by the coordinating investigators and the responsible biometrician. Reasons that may necessitate a termination of the trial include the following: It appears that patients’ enrolment is unsatisfactory with respect to quality and/or quantity or data recording is severely inaccurate and/or incomplete or any external evidence demanding a termination of the trial. In this case the independent ethics committee (IEC) will be informed.

The study design is prospective observational. No interventions are introduced or prohibited by this study. No randomization will be carried out.

### 2.1 Outcomes/endpoints

For the PRIO-Registry, data about demography, socioeconomic status, life style properties, cancer disease and therapy, IOM and patient reported outcomes will be collected. IOM will be collected and adapted to the different areas of integrative oncology. Demography, cancer data and Patient reported outcomes will be collected using validated questionnaires (listed below).

Demography and socioeconomic status: Data collection about demography and socioeconomic status follow the “Demographische Standards” of the German Federal Statistical Office (10) and are adapted to the medical question of this study.

Data about oncological diagnostics and treatment: For the data of the cancer disease and its treatment, the items of the oncological basic data set of the Working Group of German Tumor Centers will be used (11). The data is collected by the CCCU mandatorily for each cancer patient. The data is taken from the general data collection for the German Cancer Registry.

Lifestyle, risk factors and habits will be assessed using a questionnaire developed by the Research Group for Integrative Medicine at the University Hospital of Ulm. The questionnaire contains 26 questions in 9 topic areas. It follows the concept of Lorenzo Cohen, dividing influencing lifestyle factors into 6 fields: Social relationships, stress, sleep, exercise, nutrition and environment (12).

IOM will be recorded following the different areas of integrative oncology: (i) lifestyle modification, (ii) mind-body practices, (iii) natural products, with a questionnaire developed at the Research Group for Integrative Medicine at the University Hospital of Ulm. In order to cover the widest possible range of measures, the questionnaire is broadly worded, refers to the various fields of integrative oncology, inquires about them and leaves the option of free text answers.

Quality of life will be assessed with the internationally validated quality of life questionnaire for cancer patients EORTC QLQ-C30 (13).

Cancer related fatigue will be assessed with the internationally validated fatigue questionnaire for cancer patients EORTC QLQ-FA12 (14).

Sleep quality will be assessed with a validated sleep status questionnaire PSQI (15).

Depression and anxiety symptoms will be recorded with a very short validated questionnaire PHQ-4 (16).

Symptoms from cancer and cancer treatment will be recorded using the *Patient-Reported Outcomes version of the Common Terminology Criteria for Adverse Events* (PRO-CTCAE^®^), which was introduced by the National Cancer Institute of the United States of America (17).

Patient satisfaction regarding oncological treatment will be assessed with the internationally validated patient satisfaction questionnaire for cancer patients EORTC PATSAT-C33 (18).

The measure yourself medical outcome profile (MYMOP2) is a validated questionnaire to measure a patient weighted symptom description and its change over time (19).

### 2.2 Patient schedule and documentation

Data will be collected for all fields during a baseline visit at the beginning of the integrative oncology consultation. Data will be collected for all fields except for basic demography and socioeconomic data on visits 3, 6 and 12 months after baseline and yearly follow-up visits. Additional visits can be carried out according to special events in the course of a patient like recurrence or death.

The patients follow the conventional medical cancer treatment, the integrative treatment is complementary.

### 2.3 Data management

All protocol-required information collected during the trial will be entered by the investigator, or designated representative, in the electronic case report forms (eCRF). Study data will be collected and managed using REDCap electronic data capture tools hosted at Ulm University (20, 21). REDCap (Research Electronic Data Capture) is a secure, web-based software platform designed to support data capture for research studies, providing 1) an intuitive interface for validated data capture; 2) audit trails for tracking data manipulation and export procedures; 3) automated export procedures for seamless data downloads to common statistical packages; and 4) procedures for data integration and interoperability with external sources. To assure a safe and secure environment for data acquired, data transmission is encrypted with secure socket layer (SSL) technology. Only authorized users are able to enter or edit data. All changes to data are logged with a computerized timestamp in an audit trail. All data will be pseudonymized. Preparations are underway for direct online data entry by study patients, to minimize the workload and make participation more attractive for cooperation partners.

### 2.4 Statistical procedures

The statistical analysis is carried out with the program R in version 4.4.2 or higher (22). Descriptive statistics will be used to describe the study population and its changes over time. Multivariate regression models will be used to describe the strength of association of clinically important variables and patient reported outcome measures with IOM data. Statistical measures will be adopted to new questions arising from topics in the field of oncology or integrative oncology.

The open text entries in the IOM questionnaire are treated as an open and growing selection list based on the measures entered. Similar entries in different formats are treated as a single choice. For reporting purposes, different choices may be grouped into IOM clusters. IOM measures, as one of the main topics of the registry, will be reported descriptively in their variety and frequency of use. Correlations of IOM with demographic or oncologic data will be explored.

#### 2.4.1 Sample size calculation

The PRIO-Registry is a clinical registry with explorative nature. Thus, no sample size calculation was performed.

#### 2.4.2 Missing data handling

For descriptive statistics, missing data will be reported as such. For statistical computations, multiple imputation techniques will be used.

### 2.5 Quality assurance and quality management

All partners will work in accordance with Good Clinical Practice Guidelines and applicable laws. Continually, trial related risks will be listed and evaluated. This risk analysis serves as basis for quality management of the trial.

The functioning of the registry will be regularly evaluated and discussed with peers to optimize the study procedures and evaluation methods.

### 2.6 Clinical data monitoring

Monitoring will be based on patient safety, patient rights, protocol adherence and data.

All investigators have to give access to trial specific patient data to monitors and agree to be visited before, during and after completion of the study to ensure that the study is conducted, recorded and reported according to the study protocol, Good Clinical Practice (GCP) requirements and the applicable laws and regulations i.e. data protection. Monitoring strategy will be a combination of centralized and onsite monitoring. On-site monitoring will focus on patient informed consent and correct and complete recording and documentation of endpoints by source data verification.

### 2.7 Assessment of safety

As no intervention is introduced by this study, no adverse events will be recorded.

### 2.8 Definition, documentation and classification of Serious Adverse Events

As no intervention is introduced by this study, no adverse events will be recorded.

### 2.9 Ethical and legal aspects

The PRIO-Registry study is conducted according to the Medical Association’s professional code (Berufsordnung der Bundesärztekammer) §15 (non-AMG/non-MPG trials). The coordinating investigator will ensure that this study is conducted in agreement with the laws and regulations of the country.

To ensure patient’s rights and safety the responsible investigator will ensure that the trial will be conducted according to the ethical principles laid out in the declaration of Helsinki.

This study protocol has been written and the study will be conducted and analyzed in accordance with ICH E6 (R2) GCP and to all relevant national and international rules and regulations.

All patients will be informed of the aims of the study, the procedures and possible hazards to which he/she will be exposed. Furthermore, it is the responsibility of the investigator to explain patients their duties within the trial. It will be emphasized that the participation is voluntary and that the patient is allowed to refuse further participation in the trial whenever he/she wants to. This will not prejudice the patient’s subsequent care. The written informed consent form will be signed and personally dated by the patient according to the ICH guidelines on GCP. All patients will have sufficient time to decide upon the participation in this trial. One copy of the patient informed consent form will be handed out to the patient. In case of withdrawal of their informed consent, patients will be asked whether the data recorded up to that date may be used in the analysis of the trial or if it should be discarded.

Patients will be informed as to the strict confidentiality of their data, but that their medical records may be reviewed for trial purposes by authorized individuals other than their treating physician. It is the responsibility of the investigator to maintain patients’ confidentiality. During the trial, patients will be identified solely by means of their individual identification code. Trial specific documents will be stored in accordance with local data protection law/ICH-GCP Guidelines and will be handled in strictest confidence. The study center will maintain a personal subject identification list (screening numbers with the corresponding subject names) to enable records to be identified. The patients’ data will be documented in a pseudonymized form in the eCRF. Names and all confidential data of participating patients will be handled in line with the obligations of medical secrecy, the European General Data Protection Regulation (Datenschutzgrundverordnung; DSGVO), the Federal Data Protection Act (Bundesdatenschutzgesetz) and of the state Data Protection Act (Landesdatenschutzgesetz). Participating patients’ data will be recorded only in pseudonymized form. Third parties have no access to original documents. After completion of the trial, data collected during the study will be kept on file for 10 years.

The investigator will ensure that all trial-related personnel are adequately informed about the protocol, any amendments to the protocol, the investigational treatments, and their trial-related duties and functions.

The investigator will maintain a list of sub-investigators and other appropriately qualified persons to whom he or she has delegated significant trial-related duties.

According to § 15 of the Professional Code for Physicians in Germany (Berufsordnung für die in Deutschland tätigen Ärztinnen und Ärzte), the coordinating investigator will consult the Ethics Committee (EC) of the University of Ulm before the start of the trial. Once the trial has started, any changes have to be documented in a written amendment. They should be restricted to exceptional cases. Amendments then become part of the clinical trial protocol. The EC will be informed of all subsequent protocol amendments in order to determine whether formal approval must be sought and whether the informed consent document should also be revised. The EC will be informed of the end of the trial by the coordinating investigator.

### 2.10 Data sharing

An anonymized data set of each analysis will be shared after publication of the results of the respective analysis. Anonymization will be carried out by deletion or grouping of variables which are likely to allow identification of individual trial participants.

### 2.11 Financial aspects and feasibility

The current pilot phase of the registry is funded by the Software AG Foundation, Darmstadt, Germany, as part of its support for the Department of Integrative Medicine in the Department of General Surgery at Ulm University Hospital. Applications for funding from private foundations and public funds are being prepared for the next project phases.

Overall, care was taken during project planning to keep costs to a minimum. This included, in particular, the use of existing high-quality data of the included patients from the national cancer registry. All other data is only entered by the patients; as soon as it is possible for them to make entries directly in the electronic data system, the effort for the study staff is also minimized here.

The use of RedCap, an electronic data system that does not require license fees, has been demonstrated to be effective in previous registries. Furthermore, it allows for multi-center collaboration and direct data entry by patients.

## 3 Discussion

Integrative oncology is an emerging field often sought by patients. The use of IOM goes far beyond what has been researched in clinical studies and what is recorded in the scientific literature. IOM are often not mentioned by patients to their treating physicians (23), although it might be relevant regarding the individual oncological course.

With the PRIO registry we further explore the field of integrative oncology. We want to establish a solid data base to estimate the impact of integrative oncology, the methods used, and the demographic characteristics of the patients using it. We want to create more transparency in the use of IOM also to inform about possible interactions with conventional therapies and possible influence of IOM on patients in clinical trials of conventional treatments.

In Germany and other countries, cancer centers are required to submit patient data to national cancer registries. With this official registry data, the addition of the PRIO registry is an easy option to build a solid data basis about IOM use and allows comparison of populations. Leveraging existing data and adding only patient-entered data minimizes the burden on study teams and provides an easily scalable solution for other study sites. However, patient-reported data can also introduce bias, as there is no medical expert review of the data before it is entered.

With a future expansion of the registry to other centers in Germany and later internationally, additional questions will play an important role such as: How does the use of IOM differ in different centers, regions and countries? Do the demographic characteristics of IOM users differ between regions and countries?

The PRIO registry also includes validated patient reported outcome measures (PROMS). PROMS are increasingly used and valued as measures for health benefit for the individual patient and the health care system (24, 25). With these PROMS, parameters of quality of life and satisfaction with the health services and IOM will become analyzable.

Regarding data collections instruments, in the PRIO registry we focused on validated questionnaires and oncology specific instruments. The EORTC QLQ-C30 was selected as the most appropriate tool for this study, as it encompasses a broader range of quality of life dimensions in cancer patients than other questionnaires, such as the FACT-G (26). For some areas, our team developed new instruments, such as the lifestyle questionnaire and the IOM questionnaire. For lifestyle, we wanted to include all aspects that influence the course of cancer and quality of life. Existing instruments for single aspects of lifestyle, such as diet (27) or physical activity (28), either did not cover all the aspects we felt were needed in our integrative oncology counselling, or were long and too detailed and like that too burdensome for the patients. Regarding IOM, we wanted the instrument to be as sensitive as possible, to capture all possible IOM used by patients. The I-CAM-Q has been developed for complementary and alternative medicine use (29); however, the shortcomings of the questionnaire in not capturing all possible IOMs have been noted in similar registries (30).

Registries in integrative oncology can be found in the current literature and are currently running in Germany (30, 31), Switzerland (32) and the United States of America (33). However, these registries focus on one center (32), one cancer type (30), one type of IOM (31) or on evaluating the treatment of the participating sites (33). The PRIO registry will be a IOM registry on a broader scale to evaluate the impact of Integrative Oncology in oncology, its use, its trends and correlations of IOM to health outcomes. As the PRIO registry expands, international data on cancer and IOM will also be collected, as IOM use and trends differ between different world regions (34). Evaluation of these data may also support the inclusion of some IOM data items into national cancer registries.

Shortcomings and challenges of such a registry include the need for ongoing data collection, database maintenance, and analysis, which are resource intensive. Without a clear funding strategy, it may be difficult to maintain and expand the registry over time. The results of a registry study are often limited to the participating clinics or patient populations and, in developing areas of medicine, to the time of data collection. The generalizability of the results may suffer, especially if the study is multicenter and international. Differences in health care between countries may also affect the transferability of results.

Regarding future research, the data from PRIO will provide a database of correlations between IOM use and oncology data, survival data, and parameters of quality of life and satisfaction. These correlations could inform future IOM research projects on which methods appear to have an impact on cancer patients.

## Data Availability

The original contributions presented in the study are included in the article/supplementary material. Further inquiries can be directed to the corresponding author.
